# Efficient Scavenging of TEMPOL Radical by Ascorbic Acid in Solution and Related Prolongation of ^13^C and ^1^H Nuclear Spin Relaxation Times of the Solute

**DOI:** 10.3390/molecules29030738

**Published:** 2024-02-05

**Authors:** Václav Římal, Eleonora I. Bunyatova, Helena Štěpánková

**Affiliations:** 1 Faculty of Mathematics and Physics, Charles University, V Holešovičkách 2, 18000 Prague 8, Czech Republic; stepanko@mbox.troja.mff.cuni.cz; 2 Joint Institute for Nuclear Research, 141980 Dubna, Russia; bunyatel@jinr.ru

**Keywords:** TEMPOL, radical scavenging, NMR, nuclear spin relaxation

## Abstract

Dynamic nuclear polarization for nuclear magnetic resonance (NMR) spectroscopy and imaging uses free radicals to strongly enhance the NMR signal of a compound under investigation. At the same time, the radicals shorten significantly its nuclear spin relaxation times which reduces the time window available for the experiments. Radical scavenging can overcome this drawback. Our work presents a detailed study of the reduction of the TEMPOL radical by ascorbic acid in solution by high-resolution NMR. Carbon-13 and hydrogen-1 nuclear spin relaxations are confirmed to be restored to their values without TEMPOL. Reaction mechanism, kinetics, and the influence of pD and viscosity are thoroughly discussed. The detailed investigation conducted in this work should help with choosing suitable concentrations in the samples for dynamic nuclear polarization and optimizing the measurement protocols.

## 1. Introduction

Nuclear magnetic resonance (NMR) spectroscopy and magnetic resonance imaging (MRI) are among the main experimental methods for the non-destructive investigation of organic molecules in both in vitro and in vivo. One of the strategies to increase the sensitivity of NMR by several orders of magnitude is represented by dynamic nuclear polarization (DNP). In DNP, the nuclear spin system of the compound under study is hyperpolarized by a polarization transfer from the spin polarization of unpaired electrons in a polarizing agent (PA) added to the sample, irradiated by microwaves [1,2]. Solid-state polarization exceeding 90% or 70% of all ^1^H or ^13^C nuclear spins, respectively, can be obtained in magnetic fields of 3–10 T at a temperature of 1–2 K [3,4,5]; the isotopes with low gyromagnetic ratios can profit from cross polarization from ^1^H [6]. The often-used PAs are the simplest stable nitroxyl radicals (NR), e.g., (2,2,6,6-tetramethylpiperidine-1-yl)oxyl (TEMPO), 4-hydroxy-TEMPO (TEMPOL), and other piperidine-series nitroxyl radicals with substituents in position four of the piperidine heterocycle. The steric effects of the four methyl groups in NRs stabilize these radicals by suppressing the reactivity of the unpaired electron [7]. NRs also benefit from their relatively low cost, commercial availability, and effectiveness as PAs [8]. Without affecting their unpaired electrons, NRs can be further modified or covalently bound to another molecule, macromolecule, or a material based on them. Other frequent PAs include trityl-derived radicals [9,10,11] or chelates with paramagnetic metal centres [12].

Dissolution DNP (dDNP) is a way of rapidly bringing the high polarization achieved in the solid state into a liquid phase [5,13,14,15,16]. Proton polarization in liquid water routinely reaches an order of 10% [17], but a polarization above 70% has been reported using UV-generated radicals [18]; ^13^C polarization levels above 30% in solution have been achieved [8]. Apart from the use of hyperpolarized molecules in high-field NMR spectrometers, a benchtop arrangement has also been developed [19]. Solutions with hyperpolarized nuclei can also be injected into biological objects placed in an MRI tomograph [20,21] and the metabolism of simple organic molecules, such as glucose [22,23], pyruvate [24,25,26], acetate [27,28,29], or others [30,31,32,33] can be observed. 

After turning off the microwaves driving the polarization transfer, the hyperpolarized nuclei undergo relaxation towards a thermodynamic equilibrium corresponding to the magnetic field and temperature at a given moment. Therefore, the nuclear spin relaxation before, during, and after dissolution decreases the signal intensity in dDNP [13]. While technical solutions have been invented to speed up the dissolution and delivery of the sample to the NMR spectrometer or MRI tomograph [34,35,36] and the contribution of the nuclear dipolar interaction with the solvent to the relaxation is minimised by the use of deuterated solvents in dDNP, which dilutes the ^1^H spins [37], the PA first used for hyperpolarization now unfavourably enhances the relaxation due to its paramagnetism. When the paramagnetic relaxation is the dominant relaxation pathway, the paramagnetic relaxation enhancements of longitudinal and transverse relaxation rates *R*_1_ = *T*_1_^−1^ and *R*_2_ = *T*_2_^−1^ (*T*_1_ and *T*_2_ are the longitudinal and transverse relaxation times, respectively) are directly proportional to the concentration of the radicals in dilute solutions [38,39]. Relaxivities *r*_1_ and *r*_2_ are defined as increase in *R*_1_ and *R*_2_ per radical concentration, respectively. Various methods for reducing the paramagnetic relaxation have been proposed. In the solid-state, spin-diffusion-relayed DNP increases the distance between the hyperpolarized nuclei and the PA [40], long-lived singlet states are created [41,42,43], or photo-induced labile radicals are quenched [44]. Magnetic fields applied along the transport pathway reduce the paramagnetic relaxation during the sample transfer [36,45].

**Figure 1:**
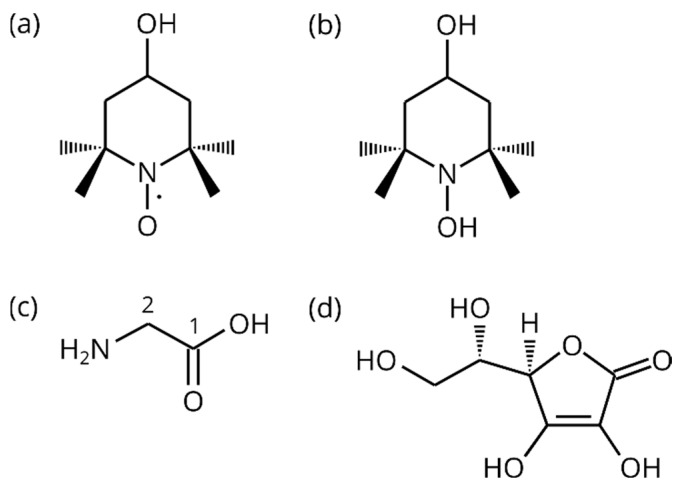
Schemes of (**a**) TEMPOL; (**b**) TEMPOL-H; (**c**) glycine with carbon numbering; and (**d**) ascorbic acid (AA).

During or shortly after the dissolution, there are several possibilities to remove the paramagnetic compounds from the sample (it should also be noted that some free radicals are toxic, and their presence in vivo is not desirable in clinical studies). Besides the quenching of photo-induced radicals mentioned above [46,47,48], hybrid organosilica materials with bound radicals can be filtered out [49,50]. A porous template of a silicon-based solid [51], thermo-responsive hydrogels that expel the hyperpolarized molecules from the radical-bearing polymer network [52], or the extraction of the radical to another solvent [53] were also used to separate the radicals from the solution. In this work, we focus on the scavenging of the radicals by L-ascorbic acid (AA or H_2_Asc, Figure 1): a chemical reaction between an NR and ascorbate anion produces a diamagnetic derivative of the nitroxide, leading to a prolongation of the nuclear relaxation times of a solute [54].

Chemical reactions that result in radical scavenging into a diamagnetic compound decrease the nuclear spin relaxation rates in the molecule under study [54,55]. A suitable reducing agent for NR is L-ascorbic acid [56]. In [54], sodium ascorbate (vitamin C or NaHAsc) was used to scavenge TEMPOL. The gradual reduction of TEMPOL was detected and an increase in the ^1^H NMR signal from the four methyl groups of the diamagnetic hydroxylamine (HA) 2,2,6,6-tetramethylpiperidine-1,4-diol (TEMPOL-H, Figure 1) was observed. The ^1^H relaxation times in glycine and acrylic acid in aqueous solutions with TEMPOL increased after ascorbate addition. The scavenging of TEMPOL by NaHAsc led to an increase in the relaxation times of ^13^C in dDNP experiments with sodium acetate [57] and pyruvic acid [18].

The ascorbate anion, HAsc^−^, reacts with the NR by a hydrogen-atom transfer [43,58,59] that produces the corresponding HA and an ascorbyl radical, Asc^•−^ [60]:

(1) $$\mathrm{NR}+{\mathrm{HAsc}}^{-}\;\overset{k_{1}}{\to }\mathrm{HA}+{\mathrm{Asc}}^{•-},$$

The reaction follows second order kinetics, i.e.,

(2) $$\frac{d\left[\mathrm{HA}\right]}{\mathrm{dt}}{=k}_{1}\left[\mathrm{NR}\right].$$

The rate constant *k*_1_ for TEMPOL was measured by NMR in D_2_O (0.20 M^−1^ s^−1^ at 23 °C [54]) and by EPR in H_2_O (6.96 M^−1^s^−1^ at 25 °C [61]; 8.7 M^−1^s^−1^ at 25 °C [62]); there is a strong kinetic isotope effect [60].

The ascorbyl radical forms a dimer (${\mathrm{Asc}}_{2}^{2-}$) and rapidly decomposes into HAsc^−^ and dehydroascorbic acid (DHA) [54,63]:

(3) $${\mathrm{Asc}}_{2}^{2-}+H^{+}\overset{k_{2}}{\to }{\mathrm{HAsc}}^{-}+\mathrm{DHA}.$$

HAsc^−^ can re-enter the reaction with NR (1), leading to a total 2:1 stoichiometry of NR:HAsc^−^ under this model, taking Equations (1) and (3) into account. DHA further decomposes by a complex cascade of reactions [64,65,66], during which a partial regeneration of HAsc^−^ can occur as well [67]. A complete reaction mechanism was proposed for the scavenging of NRs containing five-membered rings by AA, including radical scavenging by the oxidation products of DHA [68].

Apart from scavenging by AA, the radicals can enter a self-disproportionation reaction as well:

(4) $$\mathrm{NR}+\mathrm{NR}–H^{+}\overset{k_{3}}{\to }\mathrm{HA}+{\mathrm{HA}}^{+},$$

where NR–H^+^ is protonated NR and HA^+^ is an oxoammonium cation [69]. However, the very low pK < −5 [69,70] of TEMPO-based NR restricts the reaction (4) to extremely acidic conditions.

This work monitors the reaction of TEMPOL and ascorbic acid (AA) by NMR spectroscopy in liquid state. The radical reduction by AA results in an increase in the ^13^C and ^1^H relaxation times in a solute, choosing glycine (Figure 1) as a suitable benchmark molecule. Apart from relaxation measurements, we employ a real-time NMR analysis of the reaction progress and discuss possible mechanisms of radical scavenging. Our data combine several aspects of the process, explaining the role of pH and its change during the reaction, and pointing out the importance of excess AA over TEMPOL. The resulting relaxation and kinetic data obtained from NMR spectroscopy can be used for suggesting suitable conditions leading to efficient radical scavenging, e.g., in a dDNP setup. Based on our results, the concentrations of the radical and reducing agent can be tuned for particular requirements on the final relaxation times and the speed of radical quenching.

## 2. Results

### 2.1. Reactions Observed

**Figure 2:**
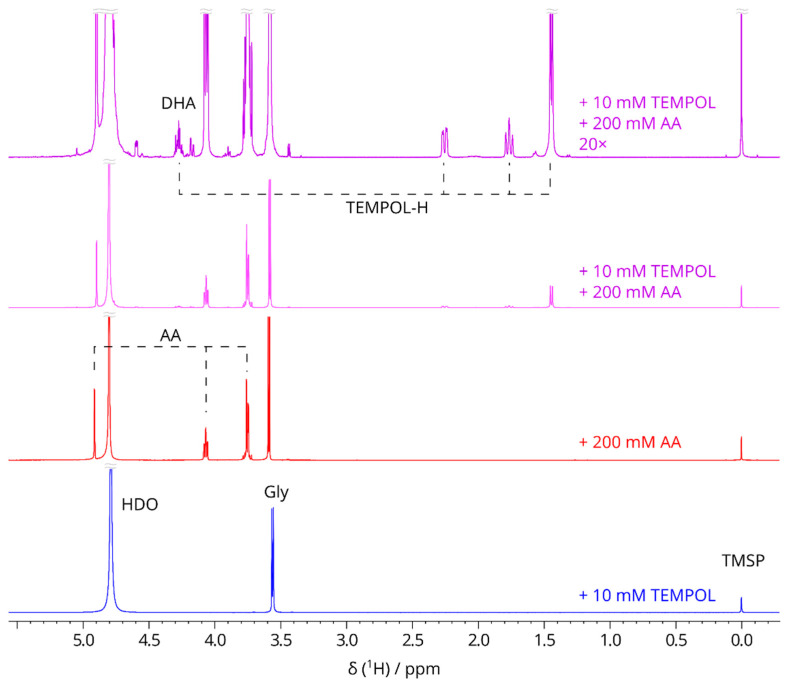
1H NMR spectra of solutions with 200 mM [1-^13^C]-glycine at 25 °C (500 MHz, 4 scans acquired). Additional solution components and scaling are indicated in the figure. The sample with TEMPOL and AA was measured more than 2 h after preparation. Major peaks: TMSP: 0 ppm; TEMPOL-H methyl protons: 1.5 ppm; glycine: doublet (caused by coupling with ^13^C) at 3.6 ppm; AA: multiplets at 3.7 ppm and 4.1 ppm and the singlet at 4.9 ppm; H4 of TEMPOL-H and DHA: multiplets at 4.3–4.4 ppm; HDO: 4.7–4.8 ppm.

^1^H NMR spectra demonstrate that the addition of AA induces the formation of TEMPOL-H with a simultaneous decrease in AA concentration and the appearance of other reaction products, namely, dehydroascorbic acid (DHA) and its descendants (Figure 2). Although there are some peak overlaps between AA and DHA in the region from 3.5 ppm to 4.0 ppm, there are several isolated peaks that allow the proper identification of the compounds and their quantification. Mainly, the signals of TEMPOL-H methyl groups, which we are the most interested in, are well separated from other resonances.

**Figure 3:**
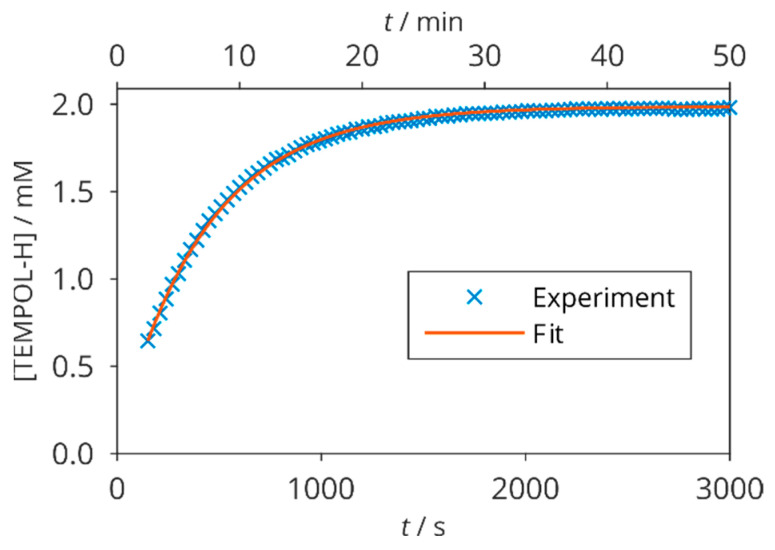
Time course of TEMPOL-H increase in 200 mM glycine, 2 mM TEMPOL, and 10 mM AA at 25 °C. The points are obtained from integral intensities of ^1^H NMR resonance of TEMPOL-H methyl groups, the solid line is fit by Equation (6).

We monitored the progress of the reaction between TEMPOL and AA by the integral intensity of the methyl signal of the diamagnetic TEMPOL-H in time. This resonance is unambiguously distinguished from the TEMPOL radical, whose signal is broadened beyond detection due to its paramagnetism. The initial rate of increase in TEMPOL-H concentration was checked in an auxiliary sample with more diluted AA (200 mM Gly + 2 mM TEMPOL + 10 mM AA). Although such TEMPOL concentration does not correspond to the usual application in DNP, we have chosen it to slow down the scavenging reaction for a better observation of its kinetics. The time-dependence of TEMPOL-H concentration, calculated from the NMR integral intensity of methyl resonances, is shown in Figure 3. As expected, it approaches the initial radical concentration for long times, and the plateau reached means that all TEMPOL is reduced by excessive AA. An exponential fit by Equation (6), provided *k*_1_ = (0.43 ± 0.02) M^−1^ s^−1^, which has the same order of magnitude as a previously published value 0.20 M^−1^ s^−1^ [54]. We ascribe the mismatch to different sample compositions and preparation protocols. Our value of *k*_1_ implies that using 200 mM AA, as in our main sample series, only 0.6% of TEMPOL remains unreacted 1 min after it is mixed with AA. Such a time scale is also well suited to dDNP experiments with delays of seconds or tens of seconds after the dissolution [14].

**Figure 4:**
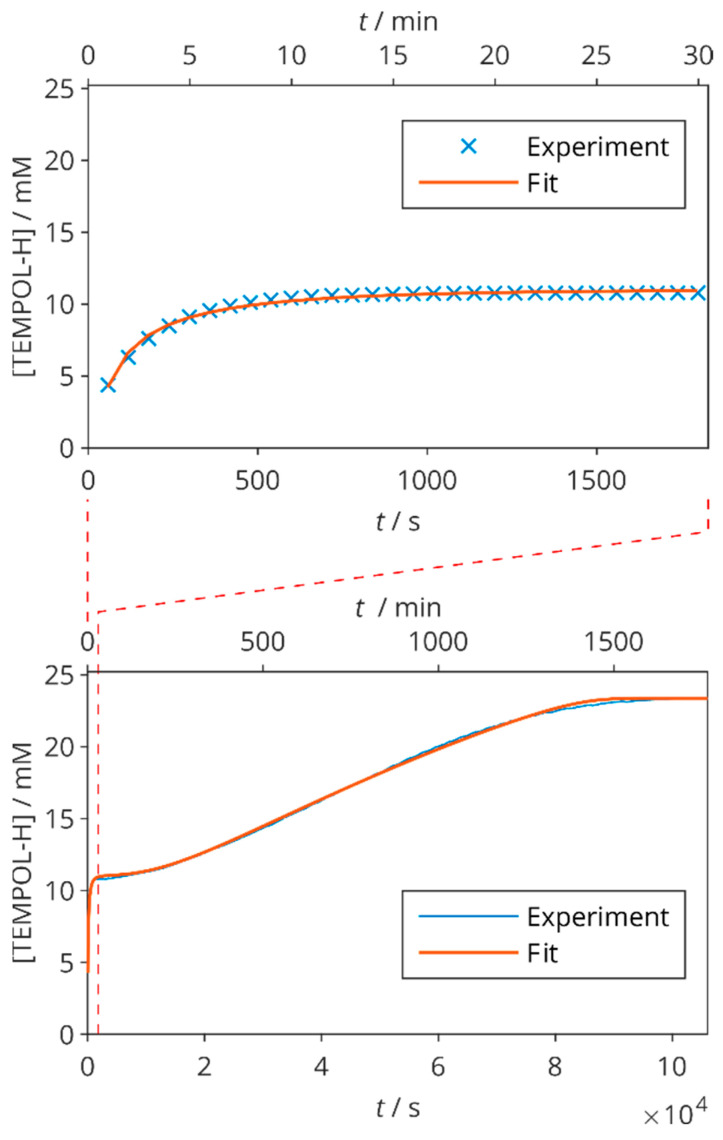
TEMPOL-H concentration in time in 200 mM Gly, 25 mM TEMPOL, and 5 mM AA at 25 °C. The points are obtained from integral intensities of ^1^H NMR resonance of TEMPOL-H methyl groups, the fits correspond to kinetic models described in Supporting Information. Top: the initial part of the reaction. Bottom: the full experimental run with the region shown in the top panel indicated by dashed lines.

When the AA concentration (5 mM) is lower than that of TEMPOL (25 mM), there is also an increase in TEMPOL-H observed up to 2[AA] after several minutes (Figure 4). This agrees with the reaction scheme (Equations (1) and (3)) and a fit including backward reactions (Supporting Information) led to *k*_1_ = (0.90 ± 0.05) M^−1^ s^−1^. More surprisingly, after some period of time (about 3000 s), a further increase in [TEMPOL-H] appears in this sample. Therefore, the plateau reached in around 600 s does not represent a true equilibrium state. Other reactions are still going on (no spontaneous formation of TEMPOL-H was observed in samples without AA). We were able to explain this behaviour only by the regeneration of AA from an unspecific DHA product Y (that might be diketogulonic acid [68], but not necessarily) by a second order reaction

(5) $$2\;Y\;\overset{k_{6}}{\to }2\;\mathrm{AA}$$

with a rate constant *k*_6_ = 0.0084 M^−1^ s^−1^ (method and further results described in Supporting Information), which was not anticipated from the literature. Although the experimental concentration in TEMPOL-H is fitted reasonably well by this model, the underlying reaction mechanism is likely to be more complex. Our data demonstrate that a tempting, but false equilibrium can persist for a relatively long time before further radical reduction takes place and that the ascorbate chemistry still deserves further research.

### 2.2. Nuclear Spin Relaxation Rates

**Figure 5:**
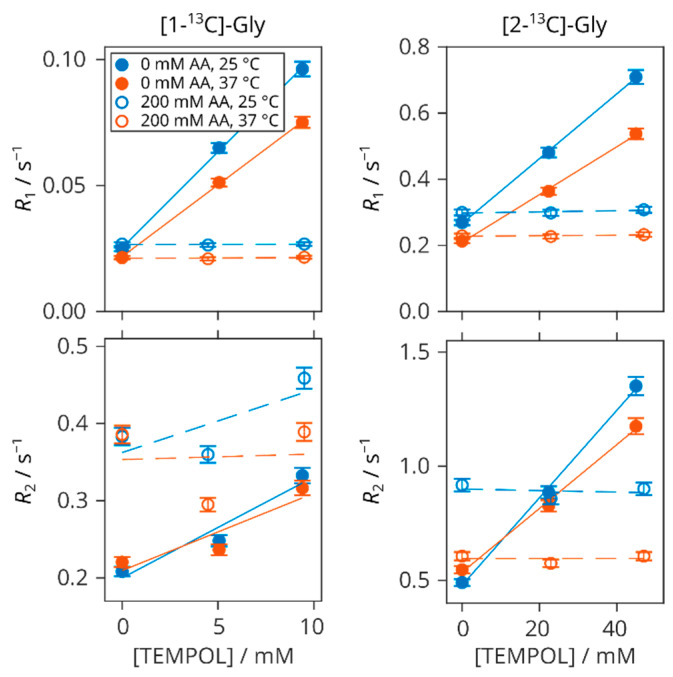
Longitudinal (*R*_1_) and transverse (*R*_2_) ^13^C relaxation rates of 200 mM [1-^13^C]- (**left**) and [2-^13^C]-glycine (**right**) versus TEMPOL concentration at 25 °C (blue) and 37 °C (red). Filled symbols: without AA; empty symbols: with 200 mM AA. Lines are linear fits.

**Figure 6:**
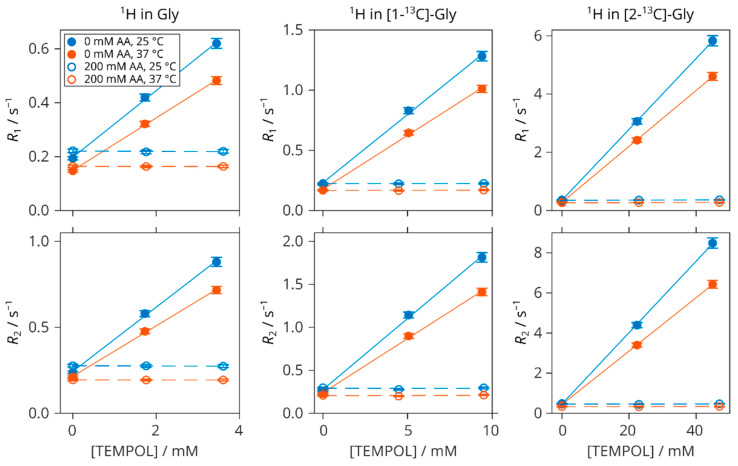
Longitudinal (*R*_1_) and transverse (*R*_2_) 1H relaxation rates in natural-abundance (**left**), [1-^13^C]- (**centre**), and [2-^13^C]-glycine (**right**) versus TEMPOL concentration at 25 °C (blue) and 37 °C (red). Filled symbols: without AA; empty symbols: with 200 mM AA. Lines are linear fits.

The ^13^C and ^1^H relaxation rates of glycine in samples without AA and with an excess of AA with respect to TEMPOL listed in Table 1 are presented in Figure 5 and Figure 6, respectively. All of the relaxation data were acquired after full radical scavenging when AA was present. It is shown how the TEMPOL addition makes the relaxation faster and that the original values in the solutions of glycine and AA without the radical are fully restored when TEMPOL is reduced by AA.

With one exception presented by the transverse relaxations of 1-^13^C, all of the relaxation rates are linear with respect to TEMPOL concentration without AA and constant when AA is added. This is also true for the ^1^H nuclei of glycine (Figure 6) and TMSP (Figure S1). Selected decay profiles are shown in Figure S2. The relaxivities obtained from the linear fits of the relaxation rates are collected in Table 2.

## 3. Discussion

### 3.1. Scavenging Mechanism and Kinetics

The decrease in AA concentration after its reaction with TEMPOL, as measured by ^1^H NMR (the differences between nominal concentrations and concentration measured after the reaction in Table 1 that are shown in Figure S3), approximately equals half of the final concentration of TEMPOL-H, which agrees well with the mechanism proposed in Equations (1) and (3). This finding, demonstrated by the high agreement between the experimental and theoretical ratio of depleted AA to TEMPOL-H concentration (Figure S3), supports previous models in a way that has not been properly discussed before.

**Figure 7:**
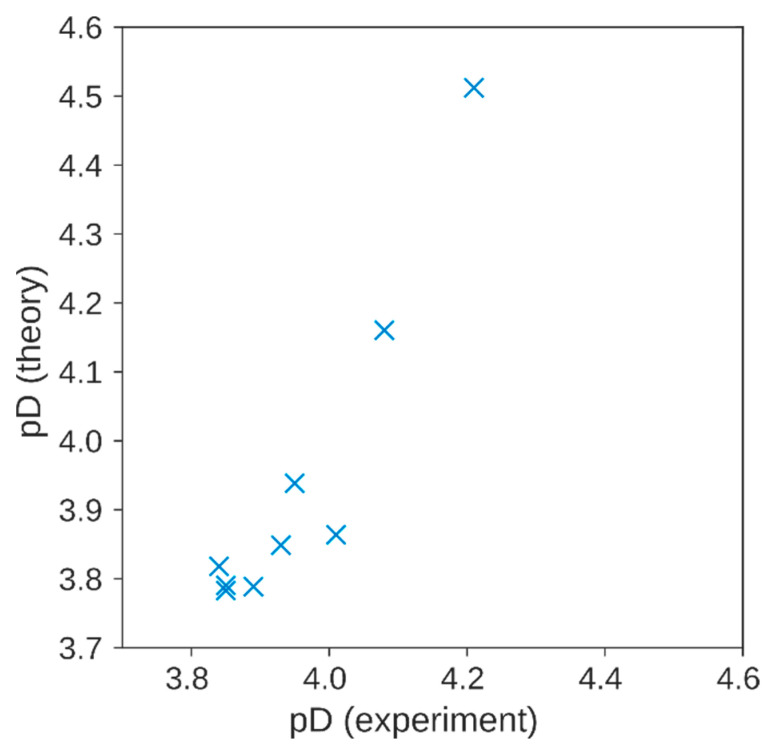
Experimental (horizontal axis) and calculated pD (vertical axis) of the solutions containing 200 mM AA and variable TEMPOL concentrations (Table 1). Method described in Supporting Information.

In addition, the measured pD of all samples is in very good accordance with the values calculated from initial concentrations and the reaction mechanism (Table 1). Figure 7 shows the correlation between calculated and experimental pD for the samples with AA. The increase in pD after TEMPOL scavenging by AA is caused by H^+^ (or D^+^) digestion during the disproportionation of the ascorbyl-radical dimer in Equation (3) and by the protonation of TEMPOL-H to TEMPOL-H_2_^+^. These effects should be kept in mind: the acidity can change during the reaction, which might have an influence when dealing with some sensitive compounds.

The kinetic modelling in Figure 4 revealed that when AA is not in an excess over the NR, a delayed reaction that leads to the further scavenging of NR must be taken into account as well. The corresponding increase in TEMPOL-H concentration is observed after the initial dose of AA is exhausted; no such behaviour can be seen when the initial AA concentration is higher than [NR]/2, because this amount is sufficient to fully reduce all of the radical as in Figure 3. Despite the care paid to sample preparation, radical scavenging by molecular oxygen can also occur [14], which would have an influence on the rate constants determined by our models.

### 3.2. Carbon Relaxations

The carboxyl ^13^C in glycine has substantially longer *T*_1_ (40 s at 25 °C) than the α carbon (3.7 s at 25 °C) because of the increased distance to the nearest hydrogen spins. On the other hand, the accessibility of both carbons to the radical paramagnetism remains similar, as shown by their relaxivities (Table 2). Therefore, the measurements of the NMR signal of the carboxyl carbon after DNP enhancement can be carried out repeatedly with small-flip-angle pulses for a relatively long time. For such purpose, the scavenging of the radical is strongly advantageous as it increases *T*_1_ four times under our conditions, having similar concentrations to what is routinely used in DNP, e.g., 40 mM TEMPOL [19], even though the dissolution dilutes the radical.

Apart from the evident relaxation enhancement by the paramagnetic molecule, there is also a strong carbon *T*_2_ relaxation shortening upon the addition of AA (Figure 5, bottom row). As opposed to the small enhancements of *R*_1_ (0.03 s^−1^ at most, Figure 5, top row), which are comparable or smaller to the values obtained for [1-^13^C]-acetate by 0.2 M AA addition [57] or pH lowering [71], the magnitude of *R*_2_ increase is comparable to the effect of the paramagnetic electrons under the concentrations used in this work.

In the search for the reasons of this strong increase in spin–spin relaxation rate, further experiments were conducted and we analysed the effects of acidity, mixed glycine isotopomers, ^1^HDO content, or the total concentration of nuclear spins. In conclusion, it is the lower pH that enhances the *R*_2_ because of the chemical exchange between the protonated and dissociated carboxyl group at pD close to the pK_GlyC_ of glycine. This explains the notable temperature dependence as well (Figure 5, bottom right).

Our relaxation experiments on the solutions containing both TEMPOL and AA were undertaken under the conditions of fully scavenged radicals. Therefore, if only a partial NR reduction is achieved at any given time during an actual dDNP process, the relaxation times shown here represent an upper limit of how they can be prolonged using the corresponding concentrations.

### 3.3. Stability in Time

NMR concentration measurements additionally conducted several days or weeks after the original experiments have shown no differences in the composition of samples except for some increase of ^1^H_2_O (~10 mM per day). The perfect stability confirms that there is no reaction in progress; it had terminated before the main measurements. Only traces of spontaneous radical reduction were found with negligible effect on the glycine ^13^C *T*_1_ and a very slow degradation of AA was observed in the time scale of months (visible also by the yellow–orange colour of the solution caused by DHA).

### 3.4. Influence of Viscosity on Relaxations

**Figure 8:**
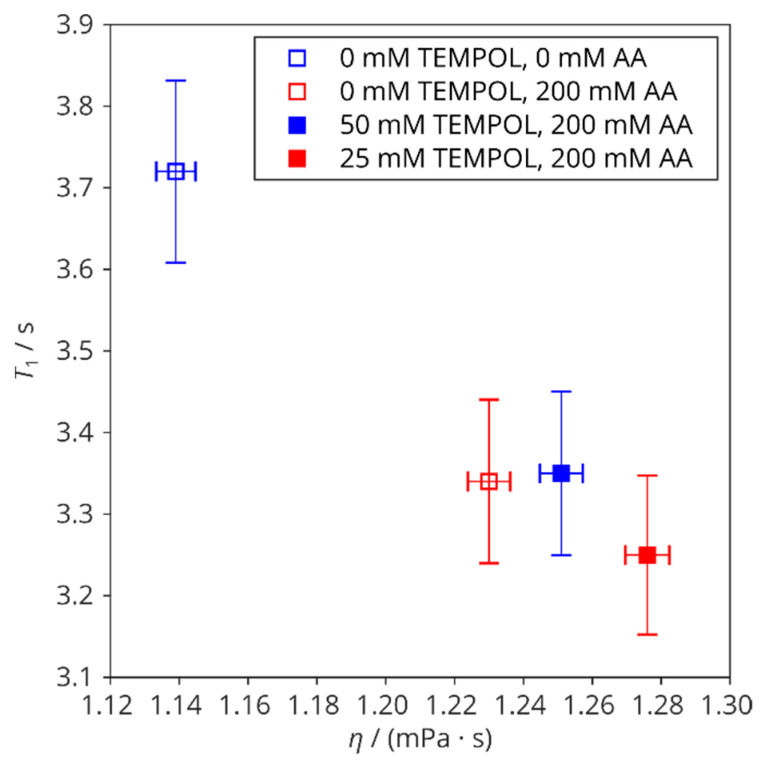
Longitudinal ^13^C relaxation times versus dynamic viscosities of mixtures containing 200 mM [2-^13^C]-glycine (25 °C). Open symbols: samples without TEMPOL; filled symbols: samples with AA and TEMPOL-H.

The addition of either AA or TEMPOL increases the dynamic viscosity, *η*, of the solution (Table 1): on average, by 0.086 mPa·s and by 0.061 mPa·s after a 200 mM AA addition at 25 °C and 37 °C, respectively. For TEMPOL addition, linear regression shows the viscosity increase 1.0 mPa·s·M^−1^ and 0.70 mPa·s·M^−1^ at 25 °C and at 37 °C, respectively. In this way, viscosity can be responsible for changes in relaxation rates in samples with AA and different TEMPOL concentrations (Figure 8). However, the scavenging of TEMPOL by AA causes a clearly distinct effect caused by the removal of electronic spins. 

### 3.5. Influence of pH on the Scavenging Reaction

We also tested the possibility that it could be the high acidity that enhances the scavenging process according to reaction (4). Experiments with DCl additions to TEMPOL instead of AA, reaching pH = 1.7, showed that this pathway proceeds only very slowly with the rate constant of TEMPOL + TEMPOL-H^+^ (using pK = −5.5 valid for TEMPO in H_2_O [70]) *k*_3_ = 3.8 × 10^−4^ s^−1^ M^−1^, close to the value 1.8 × 10^−4^ s^−1^ M^–1^ published for TEMPO + TEMPO-H^+^ in H_2_O [69]. Therefore, this reaction does not significantly contribute to our results with AA.

## 4. Materials and Methods

### 4.1. Samples

Glycine (BioUltra), [1-^13^C]- and [2-^13^C]-glycine (99% atom ^13^C), TEMPOL (97%), AA (BioXtra), D_2_O (99.9% atom D) with 0.05 wt. % 3-(trimethylsilyl)propionic-2,2,3,3-d_4_ acid, sodium salt (TMSP), and DCl (35 % in D_2_O, 99% atom D) were purchased from Sigma-Aldrich (St. Louis, MO, USA). HCl (35%, analytical grade) and NaOH (pearls, analytical grade) were purchased from Lach-Ner. Due to its better solubility than conventional TEMPO, we used TEMPOL in D_2_O. Deuterated water is used for two reasons: it has only a weak 1H signal coming from adsorbed HDO and the lack of ^1^H spins leads to slower nuclear spin relaxations of the solutes.

The stock D_2_O with TMSP was heated to 90 °C and cooled back down to ambient temperature before use to get rid of dissolved CO_2_ that would otherwise influence acidity. The solutions of TEMPOL (0.3 M) and glycine (0.6 M; non-enriched, [1-^13^C]-, and [2-^13^C]-glycine were treated separately) were mixed into individual samples to give the desired concentrations. AA solution was always prepared fresh before every sample from dry powder because of its spontaneous degradation in water [64,67,72,73]. The samples (0.6 mL) were degassed using three cycles of freeze–pump–thaw method directly in the NMR tube (5 mm Norrell S500) and sealed by krypton gas at ambient pressure.

All of the NMR samples contained 200 mM glycine (natural abundance, [1-^13^C]-, and [2-^13^C]-labelled). Samples with no TEMPOL and two samples with different TEMPOL concentrations (chosen to have reasonable effects on glycine nuclear relaxation rates) were prepared for all three glycine isotopomers used. One series of the samples was made without AA and one series with an initial 200 mM AA (Table 1). Additional samples with different concentrations were prepared in the same way for kinetic studies. 

### 4.2. NMR Spectroscopy

NMR spectra were acquired on a Bruker (Billerica, MA, USA) Avance III HD spectrometer at 11.7 T (Larmor frequency 500.13 MHz for ^1^H and 125.76 MHz for ^13^C) by a two-channel 5 mm BBFO probehead. Pulse lengths on both isotopes were adjusted from 360° nutation experiments for each sample. Chemical shifts were referenced to the internal TMSP (0 ppm). Exponential line broadening (LB = 0.5 Hz), first-order phase correction, and automatic subtraction of a fifth-order polynomial from the spectral baseline were performed in Topspin. The 1D ^1^H spectra were acquired using single-pulse excitation by 4 scans after 4 dummy scans with 30 s repetition time and 2.6 s acquisition time. The concentrations of the individual components of the samples were determined from 1D ^1^H NMR spectral integrals referenced to TMSP whose concentration was estimated as (4.15 ± 0.23) mM by auxiliary experiments (Supporting Information). Therefore, the relative error of concentrations is 5%.

Real-time ^1^H NMR data, used for the kinetic studies, were acquired as a series of single-scan 1D spectra at desired time intervals by a pseudo-2D pulse program at 25 °C.

The *T*_1_ and *T*_2_ relaxation times of ^13^C in isotopically labelled glycine and ^1^H in glycine, AA, TEMPOL-H, residual HDO, and TMSP were measured at 25 °C (the ambient temperature) and 37 °C (the temperature of the human body); the temperature was equilibrated for at least 15 min. The NMR experiments performed to detect the final effect of scavenging were started at least one hour after the mixing of all of the components of a particular sample which ensured that the reaction between TEMPOL and AA was finished. Inversion recovery (IR) and Carr–Purcell–Meiboom–Gill (CPMG) pulse sequences were employed for *T*_1_ and *T*_2_, respectively. A composite-pulse decoupling (CPD) of the non-acquired isotope (1.25 kHz waltz16 on ^1^H or 2.50 kHz garp on ^13^C for labelled glycine) was turned on during signal acquisition only. Presaturation of ^1^H was applied during the repetition delay (D1) of ^13^C CPMG. In all CPMG runs, 180-degree pulses were applied to the second channel during echoes to remove relaxations due to DD–CSA cross-correlation. Four scans were acquired for each of 10–15 relaxation delays or echo counts with repetition delays at least 5 *T*_1_ and 1.8 *T*_1_, but usually around 9 *T*_1_ and 3 *T*_1_ for IR and CPMG, respectively. The echo time in all CPMG experiments was 800 μs. Based on repeated measurements on replicated samples, the errors of relaxation times are estimated as 3%.

### 4.3. Viscosities, Densities, and pD

After all of the NMR experiments (except for the concentration determination of the TEMPOL radical) were finished, pD and viscosity were measured.

Lovis 2000 ME/DMA 4100 (Anton Paar, Graz, Austria), a combined microviscometer and density meter, was used to measure the dynamic viscosity, *η*, by falling ball principle (1.5 mm steel ball in a 1.62 mm capillary made of polychlorotrifluoroethylene, PCTFE) and density, ρ, by oscillating U-tube method at 25 °C and 37 °C [74,75]. Due to the larger volume requirements of the method for ρ (1 mL), independently prepared solutions of 200 mM glycine (without ^13^C labelling) as well as 200 mM glycine with 200 mM AA in D_2_O were used, neglecting the effect of the low concentration of TEMPOL in the main sample series (Table S1).

The pD of all samples was measured by a pH meter (Cole Parmer, Vernon Hills, IL, USA) with a calibrated micro-electrode (Hamilton, Reno, NV, USA) directly in the NMR tube and corrected from the value read on the pH meter (pH*) as pD = pH* + 0.40 [76]. Since the pD can change during the chemical reactions under study, the pD of experimentally inaccessible states were calculated from known parameters and reaction schemes as described in Supporting Information.

### 4.4. Kinetic Modelling

The least-square fits of theoretical models, described below, were applied to the experimental concentrations of TEMPOL-H in time, [TEMPOL-H] (*t*), obtained from the integral intensities of TEMPOL-H methyl resonance in the real-time pseudo-2D ^1^H NMR spectra, and were performed in MATLAB by the function lsqcurvefit.

For large excesses of AA over TEMPOL, *k*_1_ was estimated by fitting the solution of Equation (2):

(6) $$\left[\mathrm{TEMPOL}\text{-}H\right]\left(t\right)={\left[\mathrm{TEMPOL}\text{-}H\right]}_{\infty }$$

Besides *k*_1_, the optimized parameters were the final TEMPOL-H concentration [TEMPOL-H]_∞_ and the origin of the reaction Δ*t* before the start of the measurements.

More complex fits needed for the series of backward and forward reactions, including (1) and (3), and more as described in Supporting Information, involved sets of differential equations describing first and second order kinetics, analogous to Equation (2). A vector of the time derivatives of the concentrations of individual compounds was calculated and the set of ordinary differential equations was solved by the MATLAB function ode45 (Supporting Information). In this way, [TEMPOL-H] (*t*) was calculated from initial concentrations and rate constants, compared to the experimental values, and fitted by varying selected parameters as described in Supporting Information. 

All of the errors in rate constants are estimated as 5%, coming mainly from uncertainties in sample preparation.

## 5. Conclusions

The addition of TEMPOL radical causes a significant shortening of the transverse as well as longitudinal spin relaxation times of ^13^C and ^1^H nuclei in glycine. The paramagnetic relaxation enhancements are described in terms of relaxivities. Ascorbic acid restores the nuclear relaxations to the state before the introduction of the paramagnetic molecule, which proves that a full disproportionation of TEMPOL to its diamagnetic hydroxylamine occurs in the excess of AA. The radical reduction is confirmed by the measurements of the concentrations of the final products, which are in agreement with the overall reaction scheme and stoichiometry, although the kinetics indicate some further steps that have not been described yet. We support these results by monitoring the initial kinetics and agreement between experimental and theoretical pD. Apart from the presence of the paramagnetic radical, chemical exchange in glycine and solution viscosity also contribute to the nuclear spin relaxations observed.

Besides longitudinal ^13^C relaxation times, which are commonly measured in order to monitor and better understand the scavenging process, we also measured the ^1^H longitudinal as well as the ^13^C and ^1^H transverse relaxation times in solutes with variable sample composition at two temperatures. In addition, we extended the kinetic data by real-time NMR in samples with several different AA concentrations (Figure 3 and Figure 4), and a rather complex kinetics was observed.

Our results show qualitatively and quantitatively, how an addition of a radical-reducing agent (e.g., ascorbic acid as in our study) would enhance the signal-to-noise ratio in dissolution DNP. The reaction of TEMPOL with 200 mM AA has a characteristic time around 10 s, which ensures sufficiently rapid radical quenching after dissolution. Even a partial radical decrease in fast dDNP setups would be helpful to slow down the polarization decay of the hyperpolarized nuclei. More repeats of the detection phase of the experiment after a single polarisation run would be allowed by the slow longitudinal relaxation of the carboxyl carbon when the radical is scavenged (from seconds in the presence of TEMPOL to almost one minute after its reduction). The same stays valid for ^1^H experiments with even higher radical-caused relaxivities.

## Figures and Tables

**Table 1:** Composition of individual samples and concentrations measured by ^1^H NMR after the reaction. In samples without AA, the TEMPOL concentration was determined indirectly as TEMPOL-H concentration measured after an extra addition of AA (described in Supporting Information). Experimental and calculated pD and measured viscosities are also shown.

	*c*/mM										
	Nominal			Measured by ^1^H NMR				pD		*η*/mPa·s	
	Gly	AA	TEMPOL	Gly	AA	TEMPOL-H	H_2_O	exp	calc	25 °C	37 °C
unlabelled glycine	200	0	0	191			240	7.62	7.14	1.141	0.875
	200	200	0	203	200		520	3.85	3.78	1.218	0.929
	200	0	2	192		1.7	470	7.68	7.14	1.141	0.876
	200	200	2	203	195	1.8	580	3.84	3.81	1.222	0.934
	200	0	4	194		3.5	520	7.44	7.14	1.138	0.874
	200	200	4	204	195	3.6	550	3.93	3.82	1.223	0.933
[1-^13^C]-glycine	200	0	0	196			680	7.60	7.14	1.155	0.887
	200	200	0	207	199		910	3.89	3.78	1.233	0.942
	200	0	5	210		5.1	660	7.76	7.14	1.153	0.885
	200	200	5	202	193	4.5	660	4.01	3.86	1.241	0.946
	200	0	10	204		9.4	1180	7.60	7.14	1.144	0.875
	200	200	10	209	199	9.5	940	3.95	3.94	1.240	0.946
[2-^13^C]-glycine	200	0	0	200			710	7.54	7.14	1.139	0.873
	200	200	0	210	200		930	3.85	3.78	1.230	0.937
	200	0	25	199		22.4	990	7.41	7.14	1.167	0.897
	200	200	25	197	186	22.9	630	4.08	4.16	1.251	0.953
	200	0	50	202		44.9	940	7.51	7.14	1.181	0.902
	200	200	50	210	180	47	1070	4.21	4.48	1.276	0.970

**Table 2:** Relaxivities caused by TEMPOL from linear fits of relaxation rates of various nuclei. ^1^H values obtained as averages from unlabelled, [1-^13^C]-, and [2-^13^C]-glycine.

	*r*_1_/s^–1^ M^–1^		*r*_2_/s^–1^ M^–1^	
	25 °C	37 °C	25 °C	37 °C
Glycine-1-^13^C	7.7 ± 0.4	5.8 ± 0.3	12 ± 2	9 ± 2
Glycine-2-^13^C	9.7 ± 0.7	7.1 ± 0.6	19 ± 1	14 ± 1
Glycine-2-^1^H	121 ± 3	95 ± 2	179 ± 4	137 ± 2
TMSP-^1^H	209 ± 5	195 ± 4	290 ± 6	221 ± 5

## Data Availability

Data are available from the authors upon request.

## Supplementary Materials

The following Supporting Information can be downloaded at: https://www.mdpi.com/article/10.3390/molecules29030738/s1, Table S1: Densities of 200 mM natural-abundance glycine without and with 200 mM AA; Figure S1: ^1^H relaxation rates of TMSP; Figure S2: Normalized integral intensities in relaxation experiments and their fits with respect to time delay; Figure S3: Decrease of AA concentration with respect to final concentration of TEMPOL-H; Determination of concentrations by ^1^H NMR; Calculation of pD; Differential equations for kinetic models. References [77,78,79,80] are cited in the Supplementary Materials.
