# Supplementary material for: Efficient Scavenging of TEMPOL Radical by Ascorbic Acid in Solution and Related Prolongation of 13C and 1H Nuclear Spin Relaxation Times of the Solute
Source: Molecules. 2024 Feb 5;29(3):738. doi: 10.3390/molecules29030738 (PMC10856727; doi:10.3390/molecules29030738)
Supplement: Supplementary file 1 [file molecules-29-00738-s001.zip › molecules-2814641-supplementary.pdf]

Supplementary information

to

Efficient Scavenging of TEMPOL Radical by Ascorbic Acid in Solution and Related Prolongation of ^{13}C and ^{1}H Nuclear Spin Relaxation Times of the Solute

Václav Římal[a], Eleonora I. Bunyatova[b], Helena Štěpánková[a]*

[a]Faculty of Mathematics and Physics, Charles University, V Holešovičkách 2, Prague 8, Czech Republic

[b]Joint Institute for Nuclear Research, Dubna, Russia

*correspondence to: vaclav.rimal@mff.cuni.cz

Supplementary table

Table S1: Densities, ρ, of 200 mM natural-abundance glycine without and with 200 mM AA

	ρ / kg.m^{-3}	
	25 °C	37 °C
200 mM glycine	1110,9	1107,2
200 mM glycine + 200 mM AA	1122,6	1118,6

Supplementary figures

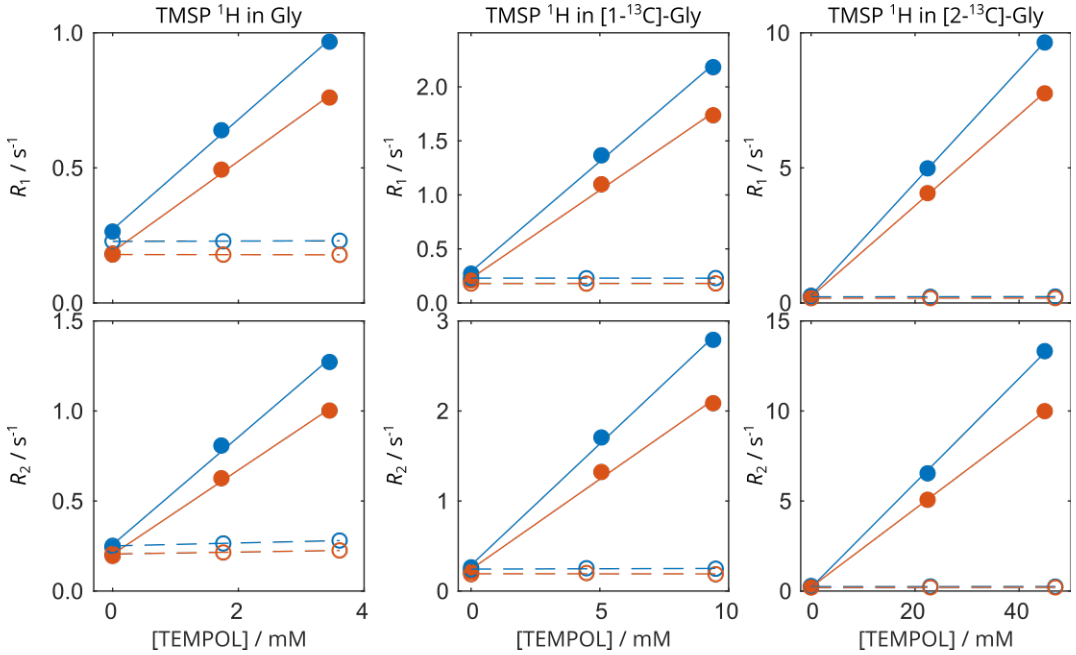

Figure S1: Longitudinal (R_1) and transverse (R_2) ^{1}H relaxation rates of TMSP in solutions with 200 mM natural-abundance (left), [1-^{13}C]- (centre), and [2-^{13}C]-glycine (right) versus TEMPOL concentration at 25 °C (blue) and 37 °C (red). Filled symbols: without AA, empty symbols: with 200 mM AA. Lines are linear fits

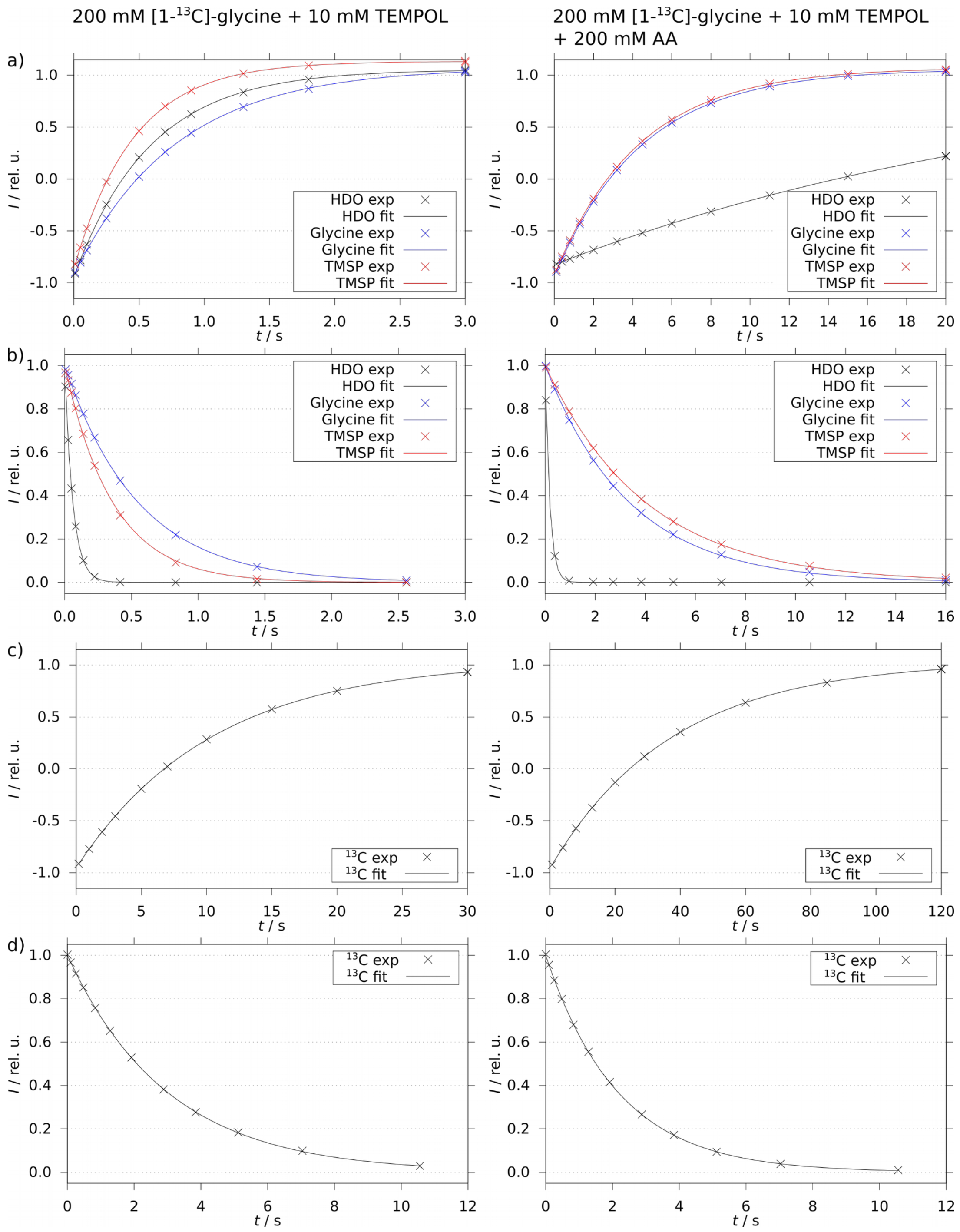

Figure S2: Normalized integral intensities, *I*, in relaxation experiments and their fits with respect to time delay *t*. Data for two samples are shown at 25 °C: 200 mM [1-^{13}C]-glycine with 10 mM TEMPOL without (left) and with 200 mM AA (right). a) ^{1}H inversion recovery for T_1 determination; b) ^{1}H CPMG for T_2 determination; c) ^{13}C inversion recovery for T_1 determination; d) ^{13}C CPMG for T_2 determination.

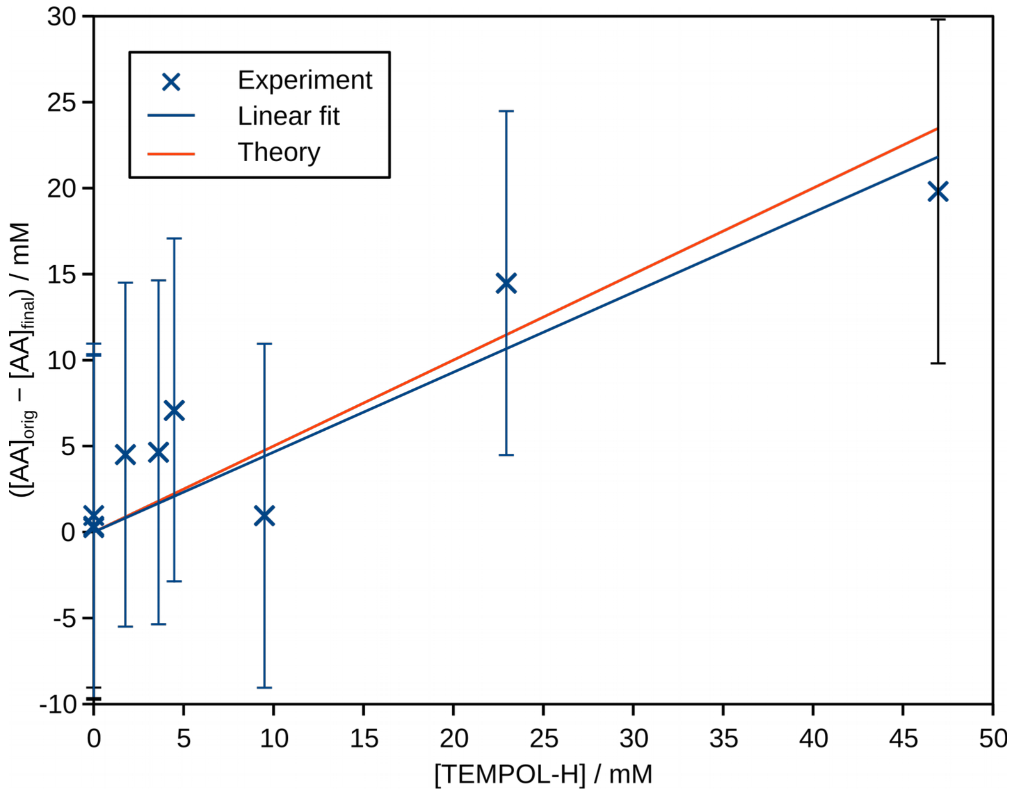

Figure S3: Decrease of AA concentration with respect to final concentration of TEMPOL-H. Crosses: experimental data from Table 1, assuming $[AA]_{orig}$ = 200 mM. Blue line: linear fit of the data with fixed zero intercept, yielding slope 0.46 ± 0.19. Red line: theoretical dependence with slope 0.5 based on reaction mechanism

Determination of concentrations by ^{1}H NMR

Concentrations of individual species in the NMR samples were measured from single-pulse ^{1}H NMR spectra with 30 s repetition delay D_1 (more than $5T_1$ of all hydrogen nuclei except HDO), 4 dummy scans, 4 recorded scans and no decoupling processed without apodization. The spectral integrals were referenced to the resonance of the three TMSP methyl groups and averaged for all non-overlapping resonances at 25 and 37 °C. The concentration of TMSP was estimated as (4.15 ± 0.23) mM by ^{1}H NMR of several solutions of precisely weighted glycine and L-leucine, which is higher than reported by the provider (0.05 wt. %, i.e. 2.90 mM).

TEMPOL concentration cannot be easily monitored by NMR due to the very short T_2 relaxation times of its nuclei. Therefore, we measured the radical concentration indirectly after it has been scavenged to TEMPOL-H. Concerning the samples containing TEMPOL without AA, 150 μl of freshly prepared 1.0 M AA in D_2O without any standard (which preserves the [TEMPOL]/[TMSP] ratio) was added directly to the NMR tube and thoroughly mixed. In this way, TEMPOL was fully reduced by a large excess of AA and the concentration of TEMPOL-H was subsequently determined as described above.

The measured integrals, I, of HDO peak were corrected according to its long T_1 relaxation time using the simple formula for I_{corr}:

$$I_{\mathrm{corr}} = \frac{I}{1 - e^{-(D_1 + t_{\mathrm{AQ}})/T_1}} \, , \tag{S1}$$

where t_{AQ} and D_1 represent the acquisition and repetition time, respectively. The same corrections made for other peaks had no effect on the final concentrations within their precision that we consider 2 % (based on analyses of different temperatures, phasing of the spectra, integration boundaries, and repetitions several days later). The final estimates of concentrations are shown in Table 2 of the main article.

Concentrations of TEMPOL-H during reaction progress were monitored from pseudo-2D real-time ^{1}H spectra with one scan at a time point.

Calculation of pD

The acidity of the solution changes during the progress of the reactions (1) and (3) described in the main text. This is caused by direct consumption of H^+ in Eq. (3) and by changes in the concentrations of dissociated and non-dissociated forms of the compounds involved. Our strategy to calculate the solution pD involves two states: the original solution of TMSP only and the situation after dissolving the other (originally neutral) compounds when their reactions are completed. The dissociation of the second proton of AA (pK > 11 [58, 61]), dissociation of DHA (pK = 9 [77]), protonation of TEMPOL radical (pK = −5.5 [70]) as well as original concentration of protonated TMSP (H−TMSP), which we estimate to have similar pK value as the one for heptanoic acid $pK_{TMSP} = 4.89$ [78], the most similar acid that we could found pK for, can be neglected at our conditions (original pD = 7.4 and pD below 5 after AA addition). Comparison of the concentrations of H^+ (isotope-independent; equivalent to H_3O^+) in the original, $[H^+]_{orig}$, and the final state, $[H^+]$, for the samples containing glycine (Gly, Gly^{N+} denoting all molecules with protonated amino group and Gly^{C-} denoting molecules with dissociated carboxyl), AA, and optionally TEMPOL yields the following equation:

$$[H^+]_{orig} = [H^+] + [DHA] + [TEMPOL{-}H_2^+] + [H{-}TMSP] + [Gly^{N+}] - [Gly^{C-}] - [HAsc^-] - [H_2O]_{dis}, \qquad (S2)$$

where, according to the reaction stoichiometry,

$$[DHA] = [TEMPOL{-}H]_{tot}/2 \qquad (S3)$$

and, coming from the dissociation and association reactions,

$$[TEMPOL{-}H_2^+] = [H^+][TEMPOL{-}H]_{tot} / ([H^+] + K_T) \qquad (S4)$$

$$[H{-}TMSP] = [H^+][TMSP]_{tot} / ([H^+] + K_{TMSP}) \qquad (S5)$$

$$[Gly^{N+}] = [H^+][Gly]_{tot} / ([H^+] + K_{GlyN}) \qquad (S6)$$

$$[Gly^{C-}] = K_{GlyC}[Gly]_{tot} / ([H^+] + K_{GlyC}) \qquad (S7)$$

$$[HAsc^-] = K_{AA}[AA]_{tot} / ([H^+] + K_{AA}). \qquad (S8)$$

The dissociation equilibrium constants of $TEMPOL{-}H_2^+$, H−TMSP, glycine amino group, glycine carboxyl, and AA are denoted K_T, K_{TMSP}, K_{GlyN}, K_{GlyC}, and K_{AA}, respectively, and the subscript "tot" indicates the total concentration of a compound regardless of its dissociation state. $[H_2O]_{dis}$ in (S2) represents water molecules which

must have dissociated in the course of the reactions; the equilibrium water ionic product, K_w, remains constant, hence

$$[H_2O]_{dis} = [OH^-] - [OH^-]_{orig} = K_w / [H^+] - K_w / [H^+]_{orig}. \quad (S9)$$

Substitution of Eqs. from (S3) to (S9) into (S2) gives a non-linear equation for $[H^+]$ which was solved numerically by function fzero in MATLAB. It was found to have a good convergence and only one solution in the relevant interval.

The result of the calculation described is, more specifically, $[D^+]$ and its negative decimal logarithm is pD for our samples in D_2O. The entering constants are taken from literature for the case of D_2O solutions if available: pK_w = 14.951 at 25 °C and pK_w = 14.616 at 35 °C [78], pK_{AA} = 4.632 [60], pK_T = 7.1 for H_2O [79], pK_{GlyC} = 2.84 [80], and pK_{GlyN} = 10.84 [80].

Differential equations for kinetic models

The data in Fig. 4 of the main article were modelled by a set of chemical reactions including (1) and (3) and their reverse (rates denoted by minus signs), Eq. (5), and the following [67]:

$$DHA + OH^- \xrightarrow{k_4} X \quad (S10)$$

$$DHA \xrightarrow{k_5} Y \quad (S11)$$

During the least-square fit to the experimental concentration of TEMPOL-H in time by function lsqcurvefit, a set of ordinary differential equations based on the reaction schemes was solved at every iteration. In addition, pD was calculated according to Eq. (S6) and following. Equilibrium constants were used to calculate the concentrations of protonated and deprotonated AA (pK = 4.632 [60]) and TEMPOL-H (pK = 7.1 [79]) as well as monomer and dimer of $Asc^{\cdot -}$ ($K_d = 10^{-3}$ M^{-1} [63]).

First, the initial part of the experimental time course up to 4000 s was fitted without reactions (6) and (S11). Fixed rates were:

k_2 $= 2.8 \cdot 10^9$ M^{-1} s^{-1} [63];

k_{-2} $= 1.1 \cdot 10^{-2}$ M^{-1} s^{-1} [68];

k_4 $= 1.83 \cdot 10^4$ M^{-1} s^{-1} (average from [67] and [77];

k_5 $= 7 \cdot 10^{-4}$ s^{-1} [68].

The results of the fit gave

$k_1 = 0.90\ M^{-1}\ s^{-1}$;

$k_{-1} = 1.11 \cdot 10^{-14}\ M^{-1}\ s^{-1}$.

In the full time window in Fig 4, only the rate constants k_4, k_5, and k_6 were optimized. The resulting curve is shown in Fig. 4 and the fitted rates are:

$k_4 = 2.60\ M^{-1}\ s^{-1}$;

$k_5 = 4.25 \cdot 10^{-5}\ s^{-1}$;

$k_6 = 8.4 \cdot 10^{-3}\ M^{-1}\ s^{-1}$.

The rate k_4 is slower than the order of $10^4\ M^{-1}\ s^{-1}$ reported previously [67,77] but we added reaction (S11) here that is responsible for DHA degradation as well. Otherwise, no AA regeneration that could explain our data would be possible.
